# Ten Years of Atypical Cartilaginous Tumors—Is Curettage Really Enough?

**DOI:** 10.3390/jcm15020457

**Published:** 2026-01-07

**Authors:** Sebastian Breden, Maximilian Stephan, Florian Hinterwimmer, Sarah Consalvo, Anna Curto Vilalta, Carolin Knebel, Rüdiger von Eisenhart-Rothe, Ulrich Lenze

**Affiliations:** Department of Orthopaedics and Sports Orthopaedics, TUM Universitätsklinikum Rechts der Isar, Ismaninger Str. 22, 81675 Munich, Germany

**Keywords:** musculoskeletal tumors, ACT, chondrosarcoma, orthopedic oncology

## Abstract

**Background**: Atypical cartilaginous tumors (ACT), formerly classified as Grade 1 chondrosarcomas (CS1) of the extremities, are hyaline cartilage-producing neoplasms. The WHO classification (4th edition, 2013) redefined ACT as locally aggressive rather than malignant tumors, prompting a shift toward less aggressive surgical management. This study reports data of a single, tertiary musculoskeletal tumor center and compares the long-term oncological outcomes of wide resections and intralesional curettage for primary ACT. **Methods**: This retrospective study included 61 patients with ACT treated at a tertiary tumor center between 2003 and 2023. Patients were divided into two cohorts: Cohort 1 was treated before 2013 with wide or radical resection, while cohort 2 was treated with an intralesional approach. Data on recurrence, revision rates, survival, and predictors of outcomes were analyzed using Kaplan–Meier survival analysis and log-rank testing. **Results**: Wide resections were performed in 24 patients, requiring prosthetic reconstruction in 76% of cases. Intralesional curettage was performed in 37 patients. Local recurrence occurred in 8% in wide resections versus 16% of curettage cases (*p* = 0.198), with no significant difference in time to recurrence between cohorts. Unplanned revision rates were higher in the wide resection group (42%) compared to curettage (35%), driven primarily by prosthesis-related complications. Overall survival was high in both groups (88% in wide resections vs. 94% in curettage; *p* = 0.705). Resection margins, and metastases were identified as significant predictors of both recurrence and tumor-related death. **Conclusions**: Intralesional curettage provides comparable oncological outcomes to wide resections with reduced morbidity, supporting its use as the preferred treatment for ACT in appropriately selected patients.

## 1. Introduction

Chondrosarcomas (CS) are the second most common primary malignant bone tumors, characterized by the production of cartilaginous matrix [1]. Among these, Grade 1 chondrosarcomas represent the least aggressive subtype and have long been a topic of extensive discussion due to their borderline biological behavior [2,3]. These tumors, typically located centrally within long bones, are often slow-growing and rarely metastasize. However, they can show local progression, causing pain or cortical destruction [3,4,5,6]. Due to this non-malignant behavior, the WHO decided in their 2013 manual on tumors of soft tissue and bone (4th edition) to define central G1 chondrosarcoma of the extremities as locally aggressive tumors and renamed it as atypical cartilaginous tumors (ACT) [1].

Traditionally, Grade 1 chondrosarcomas have been managed with wide or radical resections to ensure oncological safety [7]. This approach prioritizes complete removal of the tumor but is often associated with significant morbidity, functional impairment, and need for complex reconstructive procedures such as endoprosthetic replacements [8]. However, advances in imaging modalities, pathology, and surgical techniques have sparked a paradigm shift in the management of these tumors [1,3,4,9].

As a result, intralesional curettage, often combined with polymethyl-methacrylate (PMMA) augmentation and occasionally compound osteosynthesis, has gained traction as a treatment option for ACT [10,11]. This method offers the advantage of preserving native bone and joint function, reducing surgical morbidity, and facilitating faster recovery [10]. However, it also raises concerns about potential risks of local recurrence or inadequate oncological control.

In recent years, additional management strategies such as active surveillance have emerged for small, asymptomatic lesions with characteristic imaging features [12,13,14]. This approach aims to avoid overtreatment while maintaining close radiological follow-up. However, the criteria for selecting patients suitable for surveillance remain under debate, and robust long-term outcome data are still lacking [11,15].

Despite the diminishing use of wide resections, the evidence base comparing the long-term outcomes of intralesional versus wide/radical surgical treatments for ACT remains limited. There has been a number of retrospective evaluations of the adequacy of intralesional resection [4,9,10,16,17,18,19], but only few comparing it with wide resections directly.

In 2019, Dierselhuis et al. published a meta-analysis to determine which treatment option was superior [20]. This review included seven retrospective studies comparing wide to intralesional resections in ACT [3,12,15,21,22,23,24]. Six of these studies included relatively small patient cohorts, ranging from 23 to 39 cases, with only Campanacci et al. reporting a larger series of 85 patients [3]. A further limitation common to all comparative studies was the lack of matching between cohorts with respect to disease extent; wide or radical resections were performed exclusively in cases in whom curettage was deemed unfeasible.

The findings of the meta-analyses remained inconclusive, as Dierselhuis et al. highlighted that they could not determine whether wide resection is superior to intralesional treatment [20].

In most previous reports, patients undergoing wide resection often presented with larger or more aggressive tumors, frequently associated with higher malignant potential, whereas curettage was reserved for smaller, less extensive lesions. This inherent imbalance introduced a significant selection bias, limiting the interpretability of oncological outcomes. In contrast, our study design specifically included only those wide resections that—based on current standards—could have been treated with curettage, too.

Unlike previous comparative studies, we aimed at comparing the outcome of similar tumors (which have been treated differently though) rather than juxtapose wide resections and intralesional curettage across all cases of ACT. By restricting the analysis to this subgroup, we aimed to create a more homogeneous comparison between surgical approaches and to minimize bias related to tumor size, extent and biological aggressiveness.

## 2. Materials and Methods

### 2.1. Study Design and Patient Identification

This retrospective, single-center study was conducted at a tertiary musculoskeletal tumor center with dedicated expertise in bone sarcoma management. All patients treated between January 2003 and December 2023 for central Grade 1 chondrosarcoma (before the 2013 WHO reclassification) or atypical cartilaginous tumor (ACT; after 2013) were identified through a systematic search of the institutional tumor registry and electronic medical records. Clinical documentation, operative reports, histopathological findings, and all available radiological examinations were reviewed.

### 2.2. Inclusion and Exclusion Criteria

Patients were included if they had a histologically confirmed diagnosis of a central, low-grade chondrosarcoma or ACT located in the long bones of the extremities and had undergone surgical treatment at our institution. A minimum follow-up duration of two years was required unless local recurrence or death occurred earlier. Exclusion criteria comprised axial and pelvic lesions, high-grade chondrosarcomas, special chondrosarcoma subtypes such as myxoid, clear-cell, mesenchymal or secondary chondrosarcoma, periosteal or epiexostotic lesions, and extra-skeletal tumors. Patients treated non-operatively (watchful waiting) or those with incomplete radiological or histological documentation were excluded.

Additionally, for the comparative analysis, patients who had undergone wide or radical resection were only included if retrospective imaging review suggested that, according to current treatment standards, an intralesional approach would have been feasible. Lesions with pathological fractures, extraosseous soft tissue extension, or extensive cortical destruction were therefore excluded from the comparison cohort.

### 2.3. Radiological Evaluation and Feasibility Assessment

Tumor size was defined as the largest diameter of the lesion measured in any orientation on MRI. Endosteal scalloping was assessed using two separate parameters: (1) the degree of cortical thinning and (2) the circumferential extent of cortical thinning, defined as the proportion of the cortical circumference affected by scalloping. All measurements were performed retrospectively on preoperative imaging for both cohorts in a standardized manner.

**Figure 1:**
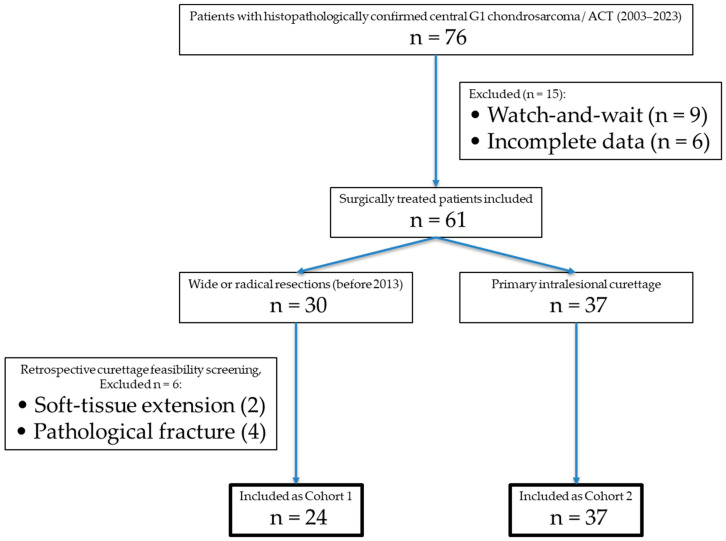
Flowchart illustrating patient selection, exclusion criteria and retrospective feasibility assessment for intralesional curettage according to contemporary radiological standards.

Curettage feasibility was defined according to the current standard at our institution for intralesional management of ACT and included: (1) purely intramedullary tumor location without complete cortical arrosion, (2) absence of an extraosseous soft-tissue component, (3) no pathological fracture, (4) endosteal scalloping involving less than two-thirds of the cortical circumference, (5) no destruction of the subchondral bone at joints and (6) absence of aggressive periosteal reaction. Lesion size alone was not considered an absolute exclusion criterion but was interpreted in conjunction with cortical integrity and soft-tissue involvement. For visualization of the inclusion process see Figure 1.

Two independent readers (one junior and one senior orthopedic tumor surgeon) performed the feasibility assessment in a blinded fashion Interobserver agreement was substantial (Cohen’s κ = 0.68; *p* < 0.001), with concordant classification in 93% of cases (28/30).

Discrepant cases were discussed in a consensus meeting. Borderline cases involved one extensive endosteal scalloping close to the two-thirds threshold, which was ultimately included and minimal cortical breakthrough without extraosseous extension, which was nevertheless excluded, as minimal soft-tissue extension could not be ruled out.

### 2.4. Surgical Treatment Before 2013: Wide or Radical Resection

Before 2013, standard treatment at our institution consisted of wide or radical resection based on oncological principles aiming for tumor-free margins. Reconstruction techniques were selected according to the location and extent of the defect. Megaprosthetic reconstruction was used in the majority of cases involving the distal or proximal femur, proximal tibia, or proximal humerus. Biological reconstruction and standard joint arthroplasty were performed in selected cases. Resection margins were classified pathologically as R0 (tumor-free), R1 (microscopically positive), or R2 (macroscopically positive).

### 2.5. Surgical Treatment After 2013: Intralesional Curettage

Following the 2013 WHO reclassification, intralesional curettage became the institutional standard for ACT of the extremities. All procedures were performed with a standardized technique, including the creation of a cortical window, careful mechanical removal of the lesion, and extended curettage with a high-speed burr to eliminate microscopic tumor remnants. Chemical adjuvants such as hydrogen peroxide were used at the discretion of the surgeon. The resulting defect was routinely filled with polymethyl-methacrylate (PMMA) cement to provide immediate stability and facilitate postoperative radiographic surveillance. When curettage resulted in a mechanically relevant cortical deficit, plate osteosynthesis was added to prevent postoperative fracture.

### 2.6. Follow-Up Protocol and Definition of Outcomes

Postoperative follow-up followed a standardized schedule, consisting of clinical and radiological evaluations every six months during the first two years and annually thereafter. Routine follow-up included MRI and plane radiographs, CT was obtained in cases where radiographic or clinical findings suggested local recurrence.

Imaging findings suggestive of recurrence included newly occurring or progressive nodular contrast enhancement within the former resection cavity or adjacent bone, progressive osteolysis, and/or new endosteal scalloping at the surgical site. In cases with high radiological suspicion for recurrence, histological confirmation was obtained by image-guided biopsy or surgical revision. In the present study, all cases of suspected local recurrence were histologically confirmed. Time to recurrence was defined as the interval between the primary surgical procedure and the date of radiological detection confirmed by histopathology.

Revision surgeries were classified as unplanned if performed due to complications or recurrence. Planned implant removals, particularly following curettage combined with osteosynthesis, were not considered revision procedures. Overall survival and tumor-specific survival were determined based on the most recent clinical documentation.

### 2.7. Histopathological Assessment

All histopathological examinations were performed by board-certified musculoskeletal pathologists at our institution. Diagnoses were established in accordance with WHO criteria and included evaluation of tumor grade, adequacy of sampling, and the presence of dedifferentiated components. For resection specimens, pathological assessment also included margin classification according to standard oncological definitions.

### 2.8. Statistical Analysis

All statistical analyses were performed using SPSS software version 29 (IBM, Chicago, IL, USA). Categorical variables were analyzed using Chi-square or Fisher’s exact tests, while continuous variables were evaluated using Student’s *t*-tests or Mann–Whitney U tests depending on data distribution. Kaplan–Meier survival analyses were conducted for recurrence-free survival, revision-free survival, and overall survival. Differences between survival curves were compared using log-rank tests.

The proportional hazards assumption for Cox regression was assessed using log-minus-log survival plots and was found to be violated for several variables. In combination with the limited number of oncological events, this was considered to preclude the use of Cox regression models. Therefore, no Cox regression and multivariate analyses are reported, and all survival comparisons are based on Kaplan–Meier estimates and log-rank testing only. A two-sided *p*-value < 0.05 was considered statistically significant.

### 2.9. Ethical Approval

The study was conducted in accordance with the principles of the Declaration of Helsinki and received approval from the Ethics Committee of the Technical University of Munich (Approval No. 2024-342-S-CB, approved on 12 March 2024). Because of the retrospective use of pseudonymized patient data, the requirement for written informed consent was waived by the ethics committee.

## 3. Results

### 3.1. Patient Characteristics

A total of 76 patients with primary, central Grade 1 chondrosarcoma of the extremities/ACT were identified between 2003 and 2023. Of these, nine were managed with a watchful-waiting approach, while 67 underwent surgical resection. Among the surgically treated cases, 30 patients received wide or radical resections, with intralesional curettage deemed feasible in 24 cases (Cohort 1). The remaining 37 patients underwent intralesional curettage (Cohort 2), see Table 1.

The final group of 61 patients with a mean age of 49 years (range: 16–81) and a male-to-female ratio of 0.6:1 (23:38). Tumors were located in the upper extremities in 33% (*n* = 20) and in the lower extremities in 67% (*n* = 41). No metastases were present at initial diagnosis. Tumor location and age were not significantly different between cohorts, but there was a significant difference in sex distribution (*p* = 0.033). Tumor size (*p* = 0.567), degree of cortical thinning (*p* = 0.203) and circumferential extent of endosteal scalloping (*p* = 0.309) did not differ significantly between the two cohorts.

### 3.2. Treatment Characteristics

Wide resections were performed in 24 patients before 2013 (see Table 1). In 18 cases (76%), megaprosthetic reconstructions were required, which included seven distal femur replacements, six proximal femur replacements, three proximal humerus replacements, as well as one proximal tibia and one total femur replacement. Biological reconstruction or standard prostheses (one knee and two hip replacements) were performed in 12%. Complete resection (R0) was achieved in 96% of cases, contaminated margins (R1) were found in 1 patient.

Intralesional resection (curettage) was performed in 37 cases (see Table 1). No patients in either cohort received systemic oncological treatment or radiation therapy.

### 3.3. Outcomes

The mean follow-up time was 93 months (138 months in C1 and 64 months in C2). The local recurrence rate in patients undergoing wide resection was 8% (2/24; one after R1 resection) after a mean of 62 months. Revision surgeries were required in 37% (9/24), predominantly due to prosthesis-related complications (70%, *n* = 7) and for resection of recurring tumors (20%, *n* = 2). Prosthesis-related complications comprised aseptic loosening in three cases, deep infection in one, periprosthetic fracture two, and polyethylene wear in one case. One patient underwent a planned implant removal after biological reconstruction (10%). The mean time to revision was 56 months. After an average follow-up of 138 months, 88% (21/24) were alive, with one tumor-related death following metastatic recurrence (upgrade to G2 tumor), one death due to infection of the mega-prosthesis and one non-tumor related death.

For intralesional resections, the recurrence rate was 16% (6/37), after a mean of 8 months. Revision surgeries were required in 11% (4/37) for second resection after local recurrence. In two cases of very small recurrences, a watch-and-wait approach was favored instead of revision surgery. Planned implant removals were performed in 9 cases (24%). After an average follow-up of 64 months, 94% (35/37) of patients were alive. One tumor-related death occurred due to rapid local recurrence with systemic spread and dedifferentiation 14 months postoperatively, the second death was not tumor related.

**Figure 2:**
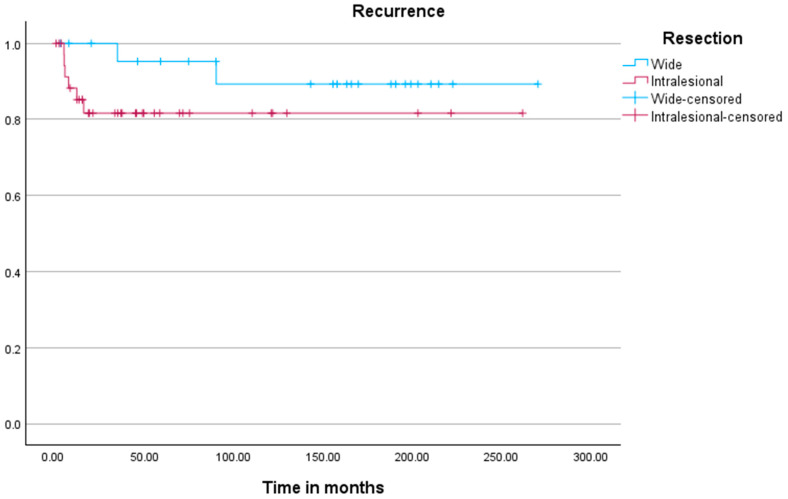
Kaplan–Meier plot visualizing the time to local recurrence between the two cohorts.

Kaplan–Meier analysis (Figure 2) revealed no significant difference in time to recurrence between both cohorts (*p* = 0.198). Kaplan–Meier analysis for local recurrence demonstrated significant associations with resection status (*p* = 0.016), tumor location (*p* < 0.001), and the development of metastases (*p* < 0.001).

**Figure 3:**
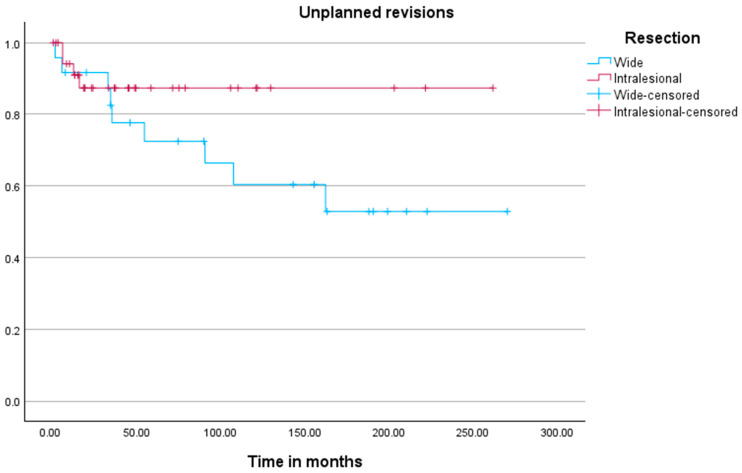
Kaplan–Meier plot visualizing the time to local unplanned revision surgeries between the two cohorts.

The analysis of time to unplanned revision surgery (Figure 3) revealed no significant difference between the cohorts (*p* = 0.150). Kaplan–Meier analysis for unplanned revision surgery identified tumor location (*p* = 0.046) and the development of metastases (*p* = 0.004) as significant associated factors.

The mean overall survival was 259 months [95% CI: 243–279], with no significant difference in tumor-related deaths between cohorts (*p* = 0.786). Univariate analysis revealed local recurrence, and development of metastases as highly significant predictors of tumor-related death (*p* < 0.0001).

To account for the marked difference in follow-up duration between cohorts, additional time-restricted survival analyses were performed with follow-up truncated at 60 months for both recurrence-free survival and overall survival. Within this uniform observation period, no statistically significant differences were observed between wide resection and curettage with regard to recurrence-free survival or overall survival (*p* = 0.110 and *p* = 0.400). These findings suggest that early oncological outcomes are comparable between both treatment strategies within the first five postoperative years.

## 4. Discussion

The management of atypical cartilaginous tumors (ACT), previously classified as Grade 1 chondrosarcomas of the extremities, has undergone a significant paradigm shift over the past decade, particularly following the 2013 WHO classification update [1]. This reclassification was caused by the locally aggressive, non-metastasizing nature of ACT and led to increased use of intralesional curettage as a surgical treatment option [2,8,12,20]. This study compared the outcome of ACTs which were treated either with curettage or wide resection.

A significant difference in sex distribution between the cohorts was observed, with a higher proportion of female patients in the curettage group. The clinical relevance of this finding remains unclear, as sex has not been consistently identified as a prognostic factor for local recurrence or survival in atypical cartilaginous tumors. Given the limited sample size and retrospective design, this difference may reflect random variation or temporal and referral-related effects rather than a true biological difference.

### 4.1. Oncological Outcome of Intralesional Curettage

Our results suggest that intralesional curettage provides adequate oncological control for most patients with ACT. The local recurrence rate in the curettage group (16%) was higher compared to the wide resection group (8%), but this difference was not statistically significant (*p* = 0.198). These findings align with previous studies that have reported recurrence rates between 10% and 20% for intralesional procedures [3,4,12,20]. The higher recurrence rate in the curettage group may be attributed to residual tumor cells left behind due to the less aggressive surgical approach, a concern that has been previously highlighted in the literature [5].

Interestingly, our Kaplan–Meier analysis showed no significant difference in ‘time to recurrence’ between the two cohorts. This suggests that, while curettage may carry a higher recurrence risk, the timing of recurrence is not significantly different compared to wide resections.

### 4.2. Revision Rates

Wide resections were associated with higher morbidity, as reflected by the 37% rate of unplanned revisions, predominantly due to prosthesis-related complications (70%). Mega-prosthetic reconstructions were performed in 76% of these cases, which often lead to long-term mechanical issues such as implant loosening, wear, or infection [7]. In contrast, the curettage group had a lower unplanned revision rate of 11%, with all unplanned revisions being performed for locally recurring tumors. This aligns with other studies that have reported improved functional outcomes and reduced surgical morbidity with intralesional approaches [9].

### 4.3. Survival Outcomes

Overall survival was high in both cohorts, with no significant difference in tumor- or treatment-related mortality (88% in the wide resection group vs. 94% in the curettage group; *p* = 0.786). Tumor-related deaths were rare, with only one event observed in each cohort. This finding reflects the generally favorable prognosis of atypical cartilaginous tumors and is consistent with previous reports [2,8,10,25].

Univariate analyses identified local recurrence and the development of metastases as factors associated with tumor-related death; however, these results must be interpreted with utmost caution given the very low number of events. It should also be noted that one death in the wide resection cohort was attributable to complications related to megaprosthetic reconstruction rather than tumor progression.

### 4.4. Oncological Limitations and Follow-Up

A relevant oncological concern after intralesional treatment of atypical cartilaginous tumors is the potential for late local recurrence and dedifferentiation. Although ACT is generally regarded as a locally aggressive but non-metastasizing entity, rare cases of dedifferentiated transformation with subsequent metastatic spread have been reported, particularly in the setting of local recurrence [11,20]. In the present cohort, only one case of dedifferentiation with rapid systemic progression occurred after curettage; however, due to the very low number of events, no reliable risk stratification is possible.

Late local recurrences have been described beyond 5 and even 10 years after initial treatment [5,11]. Given the significantly shorter follow-up in the curettage cohort, an underestimation of very late recurrences and late dedifferentiation cannot be excluded. Although additional time-restricted analyses within a uniform 5-year interval showed no differences in recurrence-free or overall survival, longer follow-up remains essential for definitive oncological risk assessment.

### 4.5. Methodological Limitations

The retrospective design and the limited cohort size, combined with the very low number of tumor-related deaths, restrict the statistical power of this study. In addition, the retrospective reassessment of historical resections carries an inherent risk of hindsight bias, as contemporary imaging interpretation and knowledge of outcomes may influence feasibility judgments despite blinded independent assessment. Owing to the limited number of oncological events, only univariate analyses were performed; consequently, all reported associations must be regarded as exploratory.

Furthermore, a temporal bias must be considered, as wide resections were predominantly performed in earlier years, whereas intralesional curettage reflects more recent practice. Advances in imaging quality, surgical training, perioperative management, and institutional protocols over time may therefore have influenced outcomes independently of the surgical technique itself.

### 4.6. Functional Outcomes and External Validity

Validated functional outcome scores such as MSTS or TESS were not systematically collected due to the retrospective design and the long inclusion period and therefore could not be analyzed. Future prospective studies should incorporate standardized patient-reported outcome measures to better quantify functional results and quality of life.

Finally, as a single-center study conducted at a tertiary sarcoma referral center, institutional expertise in surgery, imaging, pathology, perioperative management, and rehabilitation may limit generalizability. Multicenter studies including institutions with different levels of specialization are required for external validation.

### 4.7. Observational Management of ACT

An emerging topic in the treatment of ACT is the potential for a watch-and-wait approach in select cases [13,14,24]. Recent studies have suggested that non-operative management may be an option for asymptomatic patients with small, stable tumors, particularly given the slow progression and low metastatic potential of ACT [11,24,25]. All patients not treated surgically were excluded in the comparative analyses. These consisted of nine patients with cartilaginous tumors that were radiologically diagnosed as ACT but showed no clinical symptoms, that were managed with a watchful-waiting strategy. None experienced disease progression or metastasis during follow-up. This adds to the growing body of evidence supporting the consideration of non-operative management in well-selected cases, though longer follow-up and larger cohorts are necessary to validate these findings.

## 5. Conclusions

This study confirms the current practice in the treatment of ACT, demonstrating that intralesional curettage is a safe and effective alternative to wide resection for most patients. The comparable oncological outcomes and reduced surgical morbidity make curettage an attractive option, particularly for tumors in locations where functional preservation is critical. Ultimately, treatment decisions should be personalized to each patient, balancing oncological safety with quality-of-life considerations.

## Figures and Tables

**Table 1:** Comparison of baseline characteristics, treatment variables, and outcomes between patients treated with wide resection and intralesional curettage for atypical cartilaginous tumors.

		Wide Resection		Intralesional Resection		All		*p*-Value
*n*		24		37		61		
Mean age	(Years)	56		44		49		0.558
Sex	Male	13	54%	10	27%	23	38%	0.033
	Female	11	46%	27	73%	38	62%	
Mean Tumor Size		81		72		76		0.567
Cortical Thinning	none	3	12%	1	3%	4	7%	0.203
	<1/3	4	17%	3	8%	7	11%	
	1/3–2/3	12	50%	21	57%	33	54%	
	>2/3	5	21%	12	32%	17	28%	
Scalloping	none	3	12%	1	3%	4	6%	0.309
	<1/3	17	71%	23	62%	40	66%	
	1/3–2/3	4	17%	13	35%	17	28%	
	>2/3	0	0%	0	0%	0	0%	
Location	Femur	16	67%	18	49%	34	55%	0.483
	Calcaneus	1	4%	2	5%	3	5%	
	Humerus	5	21%	14	38%	19	31%	
	Tibia	1	4%	2	5%	3	5%	
	Radius	1	4%	0	0%	1	2%	
	Fibula	0	0%	1	3%	1	2%	
Mean Time to (Months)	Recurrence	248		215		230		0.198
	Unplanned Revision	175		230		198		0.150
	Tumor-related Death	261		254		259		0.786

## Data Availability

The original contributions presented in this study are included in the article. Further inquiries can be directed to the corresponding author.

## Abbreviations

The following abbreviations are used in this manuscript:

ACT	Atypical Cartilaginous Tumor
C1	Cohort 1
C2	Cohort 2
G1	Grade 1 (low-grade)
OS	Overall Survival
PMMA	Polymethylmethacrylat
R0	Complete resection
R1	Incomplete resection
WHO	World Health Organization
